# How to Cure a Felon

**Published:** 1883-06-20

**Authors:** J. M. Hole

**Affiliations:** Salem, Ohio


### HOW TO CURE A FELON.

By J. M. Hole, M. D., Salem, Ohio.

We have seen in various medical journals at different times, how to cure felons. We have never seen our cure published, and will give anybody fifty dollars who will try our cure and it fails. Well, so far so good. Now for the cure:

Take common salt, roast it on a hot stove until all the chlorine gas is thrown off, or it is all dry as you can make it. Take a teaspoonful, and also a teaspoonful of pulverized Castile soap, add a teaspoonful of Venice turpentine, mix them well into a poultice and apply to the felon. If you have ten felons at once, make as many poultices. Renew this poultice twice a day. In four or five days your felon will, if not opened before your poultice is first put on, present a hole down to the bone where the pent up matter was before your poultice brought it out. If the felonhas been cut open, or opened itself, or is about to take off the finger to the first joint, no matter, put on your poultice, it will stop it right there, and in time your finger will get well, even if one of the first bones is gone. Of course, it will not restore the lost bone, but it will get well soon. Try it.—*Peoria Med. Monthly.*

